# Supplementary material for: Comparison of methods for assessing effects of risk factors on disease progression in Mendelian randomization under index event bias
Source: Am J Hum Genet. 2026 Jun 29;113(7):1478–94. doi: 10.1016/j.ajhg.2026.06.004 (PMC13504353; doi:10.1016/j.ajhg.2026.06.004)
Supplement: Supplementary file 1 [file mmc1.pdf]

The American Journal of Human Genetics, Volume *113*

Supplemental information

Comparison of methods for assessing effects of risk factors on disease progression in Mendelian randomization under index event bias

Linxuan Zhang (张林轩), Ixavier Alonzo Higgins, Qile Dai, Apostolos Gkatzionis, Jocelyn Quistrebert, Nasir Bashir, Gopuraja Dharmalingam, Pallav Bhatnagar, Dipender Gill, Yushi Liu, and Stephen Burgess

	Positive effect											
	Confounding: -/+ (original)			Confounding: +/+			Confounding: +/-			Confounding: -/-		
Method	Median	SE	Power	Median	SE	Power	Median	SE	Power	Median	SE	Power
Ratio estimate, full sample	0.103	0.007	100.0	0.103	0.007	100.0	0.121	0.007	100.0	0.121	0.007	100.0
Ratio estimate, selected sample	0.072	0.016	98.6	0.066	0.014	99.6	0.122	0.014	100.0	0.146	0.016	100.0
Inverse-probability weighting	0.091	0.020	99.4	0.105	0.036	81.1	0.108	0.022	99.3	0.140	0.017	100.0
Slope-Hunter	0.085	0.024	71.5	0.091	0.019	72.5	0.053	0.021	59.9	0.084	0.025	64.7
Multivariable IVW (IVs only)	0.107	0.170	7.7	0.094	0.142	9.9	0.118	0.144	13.2	0.123	0.169	10.5
Corrected weighted bivariate (IVs)	0.036	1.909	3.2	0.040	0.776	7.0	0.103	0.893	11.2	0.119	1.910	5.5
Multivariable IVW (including disease)	0.105	0.018	100.0	0.106	0.016	100.0	0.119	0.016	100.0	0.122	0.018	100.0
Corrected weighted bivariate (disease)	0.106	0.017	100.0	0.107	0.015	100.0	0.120	0.015	100.0	0.122	0.017	100.0
	Null effect											
	Confounding: -/+ (original)			Confounding: +/+			Confounding: +/-			Confounding: -/-		
Method	Median	SE	Type 1	Median	SE	Type 1	Median	SE	Type 1	Median	SE	Type 1
Ratio estimate, full sample	0.000	0.007	4.9	-0.001	0.007	5.9	0.000	0.007	4.7	0.000	0.007	5.2
Ratio estimate, selected sample	-0.028	0.016	41.6	-0.022	0.014	34.5	0.021	0.014	33.5	0.031	0.016	46.1
Inverse-probability weighting	-0.016	0.020	12.7	0.008	0.032	4.9	-0.011	0.020	6.3	0.017	0.017	16.6
Slope-Hunter	0.000	0.024	52.7	0.005	0.020	53.7	-0.004	0.020	50.3	-0.001	0.025	57.6
Multivariable IVW (IVs only)	0.004	0.171	3.5	-0.004	0.142	4.3	0.005	0.144	3.4	0.007	0.173	4.4
Corrected weighted bivariate (IVs)	-0.052	2.055	2.2	-0.030	0.896	3.7	0.036	0.782	3.1	0.026	2.074	3.5
Multivariable IVW (including disease)	0.000	0.018	3.7	0.000	0.016	4.2	-0.001	0.016	3.7	0.000	0.018	3.5
Corrected weighted bivariate (disease)	0.000	0.017	7.5	0.000	0.015	7.1	-0.001	0.015	7.9	0.000	0.017	7.0

Table S1: Additional simulation results varying the direction of confounding. The leftmost columns (confounding: -/+) represents the original simulation setting. Results from Heckman's method are not provided as the method failed to converge for a non-trivial number of simulated datasets when the confounder effect on the time-to-event outcome was negative ($\delta_2 = -1$).

	Positive effect											
	Shared positive			Shared negative			Shared positive plus			Shared negative plus		
Method	Median	SE	Power	Median	SE	Power	Median	SE	Power	Median	SE	Power
Ratio estimate, full sample	0.085	0.007	100.0	0.085	0.007	100.0	0.076	0.007	100.0	0.076	0.007	100.0
Ratio estimate, selected sample	0.013	0.014	13.8	0.072	0.016	99.2	-0.005	0.014	6.2	0.038	0.015	71.3
Inverse-probability weighting	0.066	0.040	35.8	0.095	0.021	99.6	0.037	0.031	20.7	0.057	0.019	88.8
Heckman's method	-0.088	0.065	26.6	-	-	-	-0.049	0.072	12.0	-0.503	0.456	20.3
Slope-Hunter	0.078	0.019	87.4	0.071	0.025	69.5	0.068	0.018	88.5	0.070	0.022	76.1
Multivariable IVW (IVs only)	0.033	0.142	4.3	0.104	0.168	8.1	0.013	0.131	4.2	0.064	0.150	6.5
Corrected weighted bivariate (IVs)	0.007	0.925	2.0	0.053	1.861	3.3	-0.019	0.759	3.1	0.035	1.885	2.7
Multivariable IVW (including disease)	0.094	0.016	99.9	0.086	0.018	99.7	0.088	0.015	100.0	0.079	0.017	99.4
Corrected weighted bivariate (disease)	0.095	0.015	100.0	0.086	0.016	99.7	0.089	0.014	100.0	0.080	0.016	99.4
	Null effect											
	Shared positive			Shared negative			Shared positive plus			Shared negative plus		
Method	Median	SE	Type 1	Median	SE	Type 1	Median	SE	Type 1	Median	SE	Type 1
Ratio estimate, full sample	0.000	0.007	4.6	0.000	0.007	5.0	0.000	0.007	5.2	0.000	0.007	5.9
Ratio estimate, selected sample	-0.071	0.014	99.8	-0.008	0.016	6.0	-0.086	0.014	100.0	-0.039	0.015	71.8
Inverse-probability weighting	-0.021	0.038	13.7	0.013	0.021	6.1	-0.049	0.029	40.3	-0.022	0.018	21.4
Heckman's method	0.077	0.048	36.2	-	-	-	0.107	0.056	46.8	0.136	0.247	7.2
Slope-Hunter	-0.005	0.019	41.6	0.009	0.025	58.6	-0.006	0.018	41.4	0.001	0.022	51.1
Multivariable IVW (IVs only)	-0.052	0.142	5.8	0.023	0.169	3.9	-0.076	0.132	7.2	-0.008	0.151	4.5
Corrected weighted bivariate (IVs)	-0.080	0.975	5.5	-0.017	2.096	2.6	-0.082	0.918	8.0	-0.031	1.769	3.3
Multivariable IVW (including disease)	0.000	0.016	4.9	0.002	0.018	2.8	-0.001	0.016	3.2	0.001	0.017	3.9
Corrected weighted bivariate (disease)	0.001	0.015	8.9	0.002	0.017	6.5	0.000	0.015	7.3	0.001	0.016	7.8

Table S2: Additional simulation results varying the structure of confounding. Note that Heckman's method failed to converge for a non-trivial number of simulated datasets with shared negative confounding, and so results are not shown for that scenario.

	Positive effect					
	Even weaker instruments			Weakest instruments		
Method	Median	SE	Power	Median	SE	Power
Ratio estimate, full sample	0.103	0.025	96.6	0.105	0.050	56.6
Ratio estimate, selected sample	0.072	0.062	21.6	0.078	0.124	9.8
Inverse-probability weighting	0.089	0.074	21.8	0.095	0.147	9.2
Heckman's method	-0.081	0.089	15.1	-0.040	0.088	7.5
Slope-Hunter	0.082	0.065	43.1	0.082	0.103	28.4
Multivariable IVW (IVs only)	0.101	0.180	7.3	0.107	0.191	7.2
Corrected weighted bivariate (IVs)	0.046	1.897	3.4	0.066	1.650	4.3
Multivariable IVW (including disease)	0.108	0.058	45.3	0.123	0.091	27.6
Corrected weighted bivariate (disease)	0.111	0.057	48.6	0.131	0.103	26.1
	Null effect					
	Even weaker instruments			Weakest instruments		
Method	Median	SE	Power	Median	SE	Type 1
Ratio estimate, full sample	-0.002	0.025	5.9	0.002	0.050	4.9
Ratio estimate, selected sample	-0.029	0.062	6.8	-0.034	0.123	5.8
Inverse-probability weighting	-0.013	0.071	4.3	-0.017	0.141	4.3
Heckman's method	0.004	0.061	5.1	0.004	0.061	5.1
Slope-Hunter	-0.011	0.066	34.7	-0.012	0.102	20.2
Multivariable IVW (IVs only)	-0.006	0.182	4.0	0.006	0.193	3.6
Corrected weighted bivariate (IVs)	-0.019	1.897	2.8	-0.058	1.903	3.0
Multivariable IVW (including disease)	0.004	0.059	4.5	0.011	0.091	4.2
Corrected weighted bivariate (disease)	0.004	0.058	8.1	0.012	0.105	5.6

Table S3: Additional simulation results varying the strength of the genetic predictors of the risk factor. We here consider weaker instruments than in the main paper.

	Positive effect					
	50 instruments			100 instruments		
Method	Median	SE	Power	Median	SE	Power
Ratio estimate, full sample	0.103	0.003	100.0	0.103	0.002	100.0
Ratio estimate, selected sample	0.071	0.007	100.0	0.070	0.005	100.0
Inverse-probability weighting	0.091	0.010	100.0	0.091	0.008	100.0
Heckman's method	-0.266	0.045	99.4	-0.248	0.033	99.8
Slope-Hunter	0.021	0.009	72.2	0.079	0.006	81.4
Multivariable IVW (IVs only)	0.087	0.066	27.3	0.090	0.045	52.7
Corrected weighted bivariate (IVs)	-0.021	1.996	1.1	-0.077	0.919	0.9
Multivariable IVW (including disease)	0.104	0.010	100.0	0.103	0.008	100.0
Corrected weighted bivariate (disease)	0.105	0.010	99.8	0.105	0.008	100.0

	Null effect					
	50 instruments			100 instruments		
Method	Median	SE	Power	Median	SE	Power
Ratio estimate, full sample	0.000	0.003	4.5	0.000	0.002	6.2
Ratio estimate, selected sample	-0.029	0.007	98.5	-0.029	0.005	100.0
Inverse-probability weighting	-0.015	0.010	30.8	-0.015	0.009	39.4
Heckman's method	0.028	0.033	10.0	0.032	0.026	17.1
Slope-Hunter	0.012	0.009	74.1	0.008	0.006	88.5
Multivariable IVW (IVs only)	-0.006	0.067	5.4	-0.006	0.045	5.3
Corrected weighted bivariate (IVs)	-0.073	2.115	1.2	-0.082	0.835	1.1
Multivariable IVW (including GWAS)	0.000	0.010	4.2	0.000	0.008	4.7
Corrected weighted bivariate (GWAS)	0.001	0.010	5.7	0.001	0.008	7.2

Table S4: Additional simulation results varying the number of genetic predictors of the risk factor. We here consider even more instruments than in the main paper.

	Positive effect								
	Interaction for U_2			Interaction for U_1			Interactions for U_1 and U_2		
Method	Median	SE	Power	Median	SE	Power	Median	SE	Power
Ratio estimate, full sample	0.096	0.007	100.0	0.102	0.007	100.0	0.095	0.007	100.0
Ratio estimate, selected sample	0.102	0.016	100.0	0.077	0.016	99.2	0.107	0.016	100.0
Inverse-probability weighting	0.092	0.020	99.7	0.096	0.020	99.7	0.097	0.020	99.8
Heckman's method	-0.366	0.092	96.2	-0.290	0.091	86.4	-0.370	0.091	97.6
Slope-Hunter	0.124	0.024	71.1	0.087	0.024	70.7	0.122	0.024	70.6
Multivariable IVW (IVs only)	0.130	0.177	10.9	0.106	0.164	7.9	0.131	0.171	10.3
Corrected weighted bivariate (IVs)	0.050	1.954	3.4	0.048	1.987	3.0	0.079	2.039	4.6
Multivariable IVW (including disease)	0.138	0.018	100.0	0.109	0.018	100.0	0.140	0.018	100.0
Corrected weighted bivariate (disease)	0.138	0.017	100.0	0.109	0.016	100.0	0.141	0.017	100.0
	Null effect								
	Interaction for U_2			Interaction for U_1			Interactions for U_1 and U_2		
Method	Median	SE	Type 1	Median	SE	Type 1	Median	SE	Type 1
Ratio estimate, full sample	-0.001	0.007	5.9	0.000	0.007	4.9	0.000	0.007	4.6
Ratio estimate, selected sample	0.001	0.016	5.1	-0.026	0.016	36.6	0.004	0.016	4.9
Inverse-probability weighting	-0.006	0.020	5.3	-0.010	0.020	6.8	0.000	0.020	4.7
Heckman's method	-0.057	0.063	14.1	0.024	0.066	6.0	-0.059	0.063	14.1
Slope-Hunter	0.027	0.025	64.8	0.002	0.024	51.8	0.029	0.024	63.5
Multivariable IVW (IVs only)	0.021	0.178	3.5	0.006	0.166	4.2	0.022	0.170	3.4
Corrected weighted bivariate (IVs)	-0.020	2.059	1.7	-0.045	1.800	2.5	-0.008	1.977	3.3
Multivariable IVW (including disease)	0.032	0.018	38.0	0.001	0.018	3.7	0.034	0.018	42.7
Corrected weighted bivariate (disease)	0.032	0.017	47.5	0.002	0.017	7.3	0.034	0.017	50.9

Table S5: Additional simulation results adding gene–confounder interaction.

	Positive effect											
	2 additional hits			5 additional hits			10 additional hits (original)			20 additional hits		
Method	Median	SE	Power	Median	SE	Power	Median	SE	Power	Median	SE	Power
Ratio estimate, full sample	0.109	0.007	100.0	0.106	0.007	100.0	0.103	0.007	100.0	0.096	0.007	100.0
Heckman's method	-	-	-	-0.241	0.077	86.7	-0.285	0.091	85.0	-0.373	0.135	78.0
Slope-Hunter	0.016	0.023	65.5	0.084	0.023	70.9	0.085	0.024	71.5	0.089	0.022	74.4
MVIVW (including disease)	0.110	0.025	98.5	0.109	0.020	100.0	0.105	0.018	100.0	0.097	0.017	100.0
CWBLS (disease)	0.111	0.234	14.2	0.109	0.019	93.7	0.106	0.017	100.0	0.097	0.016	100.0

	Null effect											
	2 additional hits			5 additional hits			10 additional hits (original)			20 additional hits		
Method	Median	SE	Type 1	Median	SE	Type 1	Median	SE	Type 1	Median	SE	Type 1
Ratio estimate, full sample	0.000	0.007	5.0	0.000	0.007	5.2	0.000	0.007	4.9	0.000	0.007	4.6
Heckman's method	-	-	-	0.033	0.053	7.5	0.029	0.064	6.8	0.028	0.095	5.2
Slope-Hunter	0.005	0.023	61.2	0.008	0.024	58.8	0.000	0.024	52.7	-0.001	0.023	58.7
MVIVW (including disease)	-0.001	0.025	3.9	0.000	0.020	5.2	0.000	0.018	3.7	0.000	0.017	4.0
CWBLS (disease)	-0.001	0.223	0.1	0.000	0.020	7.4	0.000	0.017	7.5	0.000	0.016	8.6

Table S6: Additional simulation results varying the number of additional 'genome-wide' hits. These variants are provided to the Slope-Hunter and multivariable methods (multivariable inverse variance weighted, MVIVW; and corrected weighted bivariate least squares, CWBLS). Note that Heckman's method failed to converge for a non-trivial number of simulated datasets with 2 additional variants, and so results are not shown for that scenario.

	Positive effect											
	2 additional hits			5 additional hits			10 additional hits			20 additional hits		
Method	Median	SE	Power	Median	SE	Power	Median	SE	Power	Median	SE	Power
Heckman's	0.014	0.005	81.6	0.014	0.004	93.2	0.013	0.004	95.2	0.013	0.003	96.8
MVIVW (including disease)	0.159	0.058	75.7	0.157	0.047	91.2	0.152	0.042	94.0	0.148	0.038	96.5

	Null effect											
	2 additional hits			5 additional hits			10 additional hits			20 additional hits		
Method	Median	SE	Type 1	Median	SE	Type 1	Median	SE	Type 1	Median	SE	Type 1
Heckman's	0.000	0.005	5.1	0.000	0.004	4.8	0.000	0.004	6.8	0.000	0.003	4.7
MVIVW (including disease)	-0.001	0.061	3.9	0.001	0.049	3.8	0.001	0.044	6.3	-0.002	0.040	4.1

Table S7: Additional simulation results with a binary outcome varying the number of instruments for the disease event. These variants are provided to Heckman's method and the multivariable inverse variance weighted (MVIVW) method.

	Positive effect											
	2 additional hits			5 additional hits			10 additional hits			20 additional hits		
Method	Median	SE	Power	Median	SE	Power	Median	SE	Power	Median	SE	Power
Ratio estimate, full sample	0.109	0.007	100.0	0.107	0.007	100.0	0.103	0.007	100.0	0.096	0.007	100.0
Slope-Hunter	-0.010	0.028	53.7	-0.012	0.029	62.9	-0.039	0.032	73.1	-0.155	0.028	88.3
MVIVW (including disease)	-0.215	0.039	95.1	-0.199	0.054	93.9	-0.193	0.063	93.8	-0.185	0.066	94.9
CWBLS (disease)	-0.221	0.256	16.0	-0.202	0.056	87.8	-0.195	0.039	100.0	-0.187	0.029	99.9
	Null effect											
	2 additional hits			5 additional hits			10 additional hits			20 additional hits		
Method	Median	SE	Type 1	Median	SE	Type 1	Median	SE	Type 1	Median	SE	Type 1
Ratio estimate, full sample	0.000	0.007	4.3	0.000	0.007	5.4	0.000	0.007	3.8	0.000	0.007	4.9
Slope-Hunter	-0.026	0.032	61.0	-0.069	0.035	69.6	-0.256	0.037	82.9	-0.292	0.029	84.0
MVIVW (including disease)	-0.372	0.042	99.8	-0.345	0.058	100.0	-0.331	0.069	100.0	-0.309	0.071	100.0
CWBLS (disease)	-0.377	0.303	32.3	-0.348	0.061	96.8	-0.334	0.042	100.0	-0.312	0.031	100.0

Table S8: Additional simulation results when the additional 'genome-wide' hits have direct effects on the time-to-event outcome. These variants are provided to the Slope-Hunter and multivariable methods (multivariable inverse variance weighted, MVIVW; and corrected weighted bivariate least squares, CWBLS). Note that Heckman's method failed to converge for a non-trivial number of simulated datasets with all numbers of additional variants, and so results are not shown for that method.

Trait	Sample size	Cases	Controls	Ancestry	Reference
COVID-19 (COVID versus population)	2 942 817	159 840	2 782 977	Multi-ancestry	CHGI, 2021
COVID-19 (severe COVID versus population)	2 401 372	44 986	2 356 386	Multi-ancestry	CHGI, 2021
COVID-19 (hospitalized versus non-hospitalized)	87 833	16 512	71 321	Multi-ancestry	CHGI, 2021
Body mass index	694 649	–	–	European	Pulit et al, 2019
C-reactive protein	575 531	–	–	European	Said et al, 2022
Type 1 diabetes	520 580	18 942	501 638	Multi-ancestry	Chiou et al, 2021
Diabetic kidney disease	19 300	7 247	12 053	European	Salem et al, 2019
Hypothyroidism	1 432 752	155 096	1 277 656	Multi-ancestry	Kurki et al, 2023

Table S9: Summary of genome-wide association studies used as data sources for the summarized data analyses performed. CHGI = COVID-19 Host Genetics Initiative.

Supplementary Methods

S.1 Additional simulation study scenarios

We describe the seven additional simulation scenarios in detail here:

Direction of confounding: We generate the disease event D as a binomial random variable:

$$d_i \sim \text{Binomial}(1, \pi_i) \tag{S1}$$
$$\text{logit}(\pi_i) = -2 + 0.6x_i + \delta_1 u_{1i} + \sum_{j=1}^{J_2} \gamma_{Dj} g'_{ij}.$$

and the time-to-event outcome Y as an exponential random variable:

$$y_i \sim \text{Exponential}(\lambda_i) \tag{S2}$$
$$\log(\lambda_i) = -2 + \theta x_i + \delta_2 u_{2i} + \sum_{j=1}^{J_3} \gamma_{Yj} g^*_{ij}.$$

This is the same as the main simulation scenario, except that we originally set $\delta_1 = -1$ and $\delta_2 = +1$. We consider all positive signs of δ_1 and δ_2:

- Positive / positive (+/+): $\delta_1 = +1$ and $\delta_2 = +1$,
- Negative / positive (-/+): $\delta_1 = -1$ and $\delta_2 = +1$ (this is the original parameter setting),
- Positive / negative (+/-): $\delta_1 = +1$ and $\delta_2 = -1$,
- Negative / negative (-/-): $\delta_1 = -1$ and $\delta_2 = -1$.

Structure of confounding: In addition to U_1 and U_2 (which both affect the risk factor), we generate a third confounder U_3 with an independent standard normal distribution. We generate the disease event D as a binomial random variable:

$$d_i \sim \text{Binomial}(1, \pi_i) \tag{S3}$$
$$\text{logit}(\pi_i) = -2 + 0.6x_i + \delta_1 u_{1i} + \delta_2 u_{3i} + \sum_{j=1}^{J_2} \gamma_{Dj} g'_{ij}.$$

and the time-to-event outcome Y as an exponential random variable:

$$y_i \sim \text{Exponential}(\lambda_i) \tag{S4}$$
$$\log(\lambda_i) = -2 + \theta x_i + u_{1i} + u_{2i} + \delta_2 u_{3i} + \sum_{j=1}^{J_3} \gamma_{Yj} g^*_{ij}.$$

This is similar to the main simulation scenario, except that in the main scenario, U_1 was a confounder of the risk factor and disease event. Here, U_1 is a confounder of the risk factor, disease event, and time-to-event outcome, and U_3 is a confounder of the disease event and

time-to-event outcome (when $\delta_2 \neq 0$). U_2 remains as a confounder of the risk factor and time-to-event outcome in both cases. We consider four values for δ_1 and δ_2:

- Shared positive: $\delta_1 = +1$ and $\delta_2 = 0$,
- Shared negative: $\delta_1 = -1$ and $\delta_2 = 0$,
- Shared positive plus: $\delta_1 = +1$ and $\delta_2 = +1$,
- Shared negative plus: $\delta_1 = -1$ and $\delta_2 = +1$.

Weaker instruments: This is the same as the original scenario with 10 instrumental variants, expect that we set the mean of the distribution of the α_j parameters to 0.04 (even weaker instruments) and 0.02 (weakest instruments).

More instruments: This is the same as the original scenario with moderate strength instruments, expect that we set the number of instruments to be 50 and 100.

Gene–confounder interaction: We define g_{sum} as the sum of the instrumental variants, such that $g_{sum,i} = \sum_j g_{ij}$. We generate the exposure X as:

$$x_i = \sum_{j=1}^{J_1} \alpha_j g_{ij} + u_{1i} + u_{2i} + 0.02\delta_1 g_{sum,i} u_{1i} + 0.02\delta_2 g_{sum,i} u_{2i} + \epsilon_{Xi}. \tag{S5}$$

We consider three values for δ_1 and δ_2:

- Gene–confounder interaction for U_2 only: $\delta_1 = 0$ and $\delta_2 = +1$,
- Gene–confounder interaction for U_1 only: $\delta_1 = +1$ and $\delta_2 = 0$,
- Gene–confounder interaction for U_1 and U_2: $\delta_1 = +1$ and $\delta_2 = +1$.

Additional 'genome-wide' variants: The Slope-Hunter method requires genome-wide association data to estimate the degree of collider bias. In the original simulation, we simulated data on associations for 1000 additional variants. Associations were obtained by simulating independent beta-coefficients from a normal distribution with mean 0 and standard deviation 0.2, and their standard errors from a uniform distribution between 0.15 and 0.25.

These null associations, together with the associations for the original variants, formed the inputs for the Slope-Hunter method in the main simulation scenarios.

In an additional scenario, we varied the number of truly associated variants in the additional variants, simulating 2, 5, 10 (original), and 20 variants with a direct effect on the disease event, and the same number of variants with a direct effect on the time-to-event outcome.

Heckman's method with additional variants: Heckman's method can be implemented with either a normally-distributed continuous outcome or a binary outcome. We generated a binary outcome Y as:

$$y_i \sim \text{Binomial}(1, \lambda_i) \tag{S6}$$

$$\text{logit}(\lambda_i) = -2 + \theta x_i + u_{2i} + \sum_{j=1}^{J_3} \gamma_{Yj} g^*_{ij}.$$

This is the same form of the linear predictor as before, except that the outcome is generated using a binomial distribution rather than an exponential distribution. We note that to force the statistical package to recognize the outcome as binary, it was necessary to recode the outcome as a logical variable (`TRUE/FALSE`). We varied the number of additional variants affecting the disease event, simulating 2, 5, 10, and 20 variants with a direct effect on the disease event. For comparison, we also perform the multivariable IVW method in this scenario, using summarized data for the instrumental variants and the additional variants. In this simulation, genetic associations with the outcome are estimated using logistic regression. We note that the quantities estimated by the two methods differ, but they should be comparable in terms of power and coverage.

Additional variants affect the time-to-event outcome: In a further additional scenario, we simulated a single set of variants that affect both the disease event and the time-to-event outcome, by combining the G'_j and G^*_j variants into a single set. We considered settings with 2, 5, 10, and 20 of these variants. When included in a multivariable model, these variants would be considered as pleiotropic, as they have direct effects on the outcome that do not operate via either of the exposures (that is, via the risk factor or the disease event).

S.2 Illustrative simulation study code

```
expit <- function(x) { return(exp(x)/(1+exp(x))) }
logit  <- function(x) { return(log(x/(1-x))) }
library(survival)
library(MendelianRandomization)
library(ColliderBias)
library(SlopeHunter)
library(sampleSelection)
set.seed(496)

parts = 1e5
vars  = 10
vars_ex = 10
vars_slope = 1000
times = 100
alpha0 = 0.15 # moderate instrument setting

 ## simulation of individual-level data

 g = array(rnorm(parts*(vars+2*vars_ex)), dim=c(parts, vars+2*vars_ex))
 alpha = c(rnorm(vars, alpha0, 0.02), rep(0, 2*vars_ex))
 gamma_y = c(rep(0, vars), rnorm(vars_ex, 0.2, 0.05), rep(0, vars_ex))
 gamma_s = c(rep(0, vars), rep(0, vars_ex), rnorm(vars_ex, 0.2, 0.05))
 u1 = rnorm(parts)
 u2 = rnorm(parts)
 x  = rnorm(parts, g%*%alpha+u1+u2)
 y  = rbinom(parts, 1, expit(-2+0.6*x-u1+g%*%gamma_y))

 gscore = g%*%c(rep(1, vars), rep(0, vars_ex*2))
 s   = rexp(parts, exp(-2+0.2*x+u2+g%*%gamma_s))
 s1  = ifelse(y==1, s, NA)
 s2  = rep(1, parts)

 ## calculation of summarized data

 beta_s1 = NULL; se_s1 = NULL
 beta_x = NULL;  se_x = NULL
 beta_y = NULL;  se_y = NULL

 for (k in 1:(vars+2*vars_ex)) {
  reg_s1     = summary(coxph(Surv(s1, s2)~g[,k]))
  beta_s1[k] = reg_s1$coef[1,1]
  se_s1[k]   = reg_s1$coef[1,3]
  reg_y      = summary(glm(y~g[,k], family="binomial"))
  beta_y[k]  = reg_y$coef[2,1]
  se_y[k]    = reg_y$coef[2,2]
  reg_x      = summary(lm(x~g[,k]))
  beta_x[k]  = reg_x$coef[2,1]
  se_x[k]    = reg_x$coef[2,2]
  }

 ## naive method
```

```
cox_reg   = summary(coxph(Surv(s, s2)~gscore))  # all individuals
cox1_reg  = summary(coxph(Surv(s1, s2)~gscore)) # diseased individuals only

## bivariate method

mv1_reg     = mr_mvivw(mr_mvinput(cbind(beta_y[1:vars], beta_x[1:vars]),
     cbind(se_y[1:vars], se_x[1:vars]), beta_s1[1:vars], se_s1[1:vars]))
# instrumental variants only

mvx1_reg     = mr_mvivw(mr_mvinput(cbind(beta_y[1:(vars+vars_ex)],
     beta_x[1:(vars+vars_ex)]), cbind(se_y[1:(vars+vars_ex)], se_x[1:(vars+vars_ex)]),
     beta_s1[1:(vars+vars_ex)], se_s1[1:(vars+vars_ex)]))
# including disease event variants

## IPW method

prop_score   = glm(y~x+gscore+x*gscore, family="binomial")$fitted
ipw1_reg     = summary(coxph(Surv(s1, s2)~gscore, weights = prop_score^-1))

## Heckman's method

heck1_reg = summary(selection(y~g[,(vars+1):(vars+vars_ex)]+gscore,
                              s1~gscore))
# this only works for a normally-distributed continuous outcome or a binary outcome
# for a binary outcome, recode s1 as TRUE / FALSE

## corrected weighted least squares

summ.data1 = data.frame(xbeta=beta_x[1:vars], xse=se_x[1:vars], dbeta=beta_y[1:vars],
   dse=se_y[1:vars], ybeta=beta_s1[1:vars], yse=se_s1[1:vars])
CWBLS1 = CWBLS(summ.data1)
# instrumental variants only

summ.data1x = data.frame(xbeta=beta_x[1:(vars+vars_ex)], xse=se_x[1:(vars+vars_ex)],
   dbeta=beta_y[1:(vars+vars_ex)], dse=se_y[1:(vars+vars_ex)],
   ybeta=beta_s1[1:(vars+vars_ex)], yse=se_s1[1:(vars+vars_ex)])
CWBLS1x = CWBLS(summ.data1x)
# including disease event variants

## slope hunter

# generating simulated GWAS data

betax_s1 = rnorm(vars_slope, 0, 0.2); sex_s1 = runif(vars_slope, 0.15, 0.25)
betax_s0 = rnorm(vars_slope, 0, 0.2); sex_s0 = runif(vars_slope, 0.15, 0.25)
betax_y  = rnorm(vars_slope, 0, 0.2); sex_y = runif(vars_slope, 0.15, 0.25)

beta_s1plus = c(beta_s1, betax_s1);  se_s1plus = c(se_s1, sex_s1)
beta_yplus  = c(beta_y, betax_y);    se_yplus = c(se_y, sex_y)

dat_assocs = data.frame(beta_s1plus, se_s1plus, beta_yplus, se_yplus)
```

```
hunt1 = hunt(dat=dat_assocs, xbeta_col = "beta_yplus", xse_col = "se_yplus",
  ybeta_col = "beta_s1plus", yse_col = "se_s1plus", show_adjustments=TRUE, Plot=FALSE, M=100)

beta_s1hunt = hunt1$est$ybeta_adj[1:vars]
  se_s1hunt = hunt1$est$yse_adj[1:vars]

ivw_hunt1 = mr_ivw(mr_input(beta_x[1:vars], se_x[1:vars], beta_s1hunt, se_s1hunt))
```
